# Advancing Stroke Clinical Trials Using Community Engagement and Implementation Science Approaches

**DOI:** 10.1002/acn3.70461

**Published:** 2026-06-25

**Authors:** Lesli E. Skolarus, Devin L. Brown, Bernadette Boden‐Albala

**Affiliations:** ^1^ Department of Neurology Northwestern University Feinberg School of Medicine Chicago Illinois USA; ^2^ Department of Neurology University of Michigan Ann Arbor Michigan USA; ^3^ Department of Health, Society, and Behavior University of California, Irvine Irvine California USA; ^4^ Department of Epidemiology & Biostatistics University of California, Irvine Irvine California USA; ^5^ Department of Neurology, School of Medicine University of California, Irvine Irvine California USA

**Keywords:** clinical trial, community engagement, implementation science, stroke

## Abstract

Stroke clinical trials are essential for advancing stroke care but can face challenges with recruitment, retention, clinical relevance, and translation into real‐world practice. We propose that integrating community engagement and implementation science approaches into stroke trials can help address these needs. We conceptualize clinical trials as an evidence‐based practice and highlight that implementation frameworks linked to implementation strategies can be used to anticipate and address multilevel trial determinants. We also describe how engaging constituents across the trial lifecycle can support negotiation of inevitable trade‐offs and alignment of trial decisions with the needs, capacities, and priorities of those affected, including those responsible for implementing findings. We propose that integrating community engagement and implementation science has the potential to improve trial efficiency, strengthen relevance, accelerate translation into real‐world practice, and advance stroke health for all.

Clinical trials are essential to the advancement of stroke care. However, many stroke clinical trials are completed slower than expected, leading to delays in scientific advancement, increased resource expenditures, and, when enrollment targets are not met, reduced statistical power to detect clinically meaningful effects [1, 2, 3, 4]. In addition, clinical trials may produce results that are not applicable to routine clinical practice. For example, when trial participants do not receive all standard of care treatments such as endovascular therapy [5]. Further, uptake of practice‐changing clinical trial findings may be low, as demonstrated by the limited adoption of dual antiplatelet therapy for high‐risk TIA and minor stroke [6]. These challenges underscore the need for approaches that can improve stroke clinical trial efficiency, enhance clinical relevance, and facilitate the translation of trial findings into real‐world practice.

We propose that integrating community engagement and implementation science approaches into stroke trials may help address these needs. Despite growing interest in community engagement, evidence demonstrating the effectiveness of this approach in stroke clinical trials remains limited and has been largely restricted to primary stroke prevention [7]. Similarly, implementation science approaches have traditionally been applied to promote the uptake of guideline‐concordant treatments into routine practice, rather than to optimize the processes of clinical trials themselves [8, 9]. Yet, evidence from outside of stroke [10, 11], particularly from COVID‐19 vaccine trials [12] and public roll out efforts [13], suggests that community engagement, when paired with implementation science approaches to identify and address barriers, can improve clinical trial efficiency, quality and relevance. These observations highlight an important opportunity to re‐conceptualize stroke trials through a community‐engaged, implementation‐informed lens. In this context, we outline a pragmatic framework for integrating community engagement and implementation science across the stroke clinical trial lifecycle (Figure 1).

**FIGURE 1:**
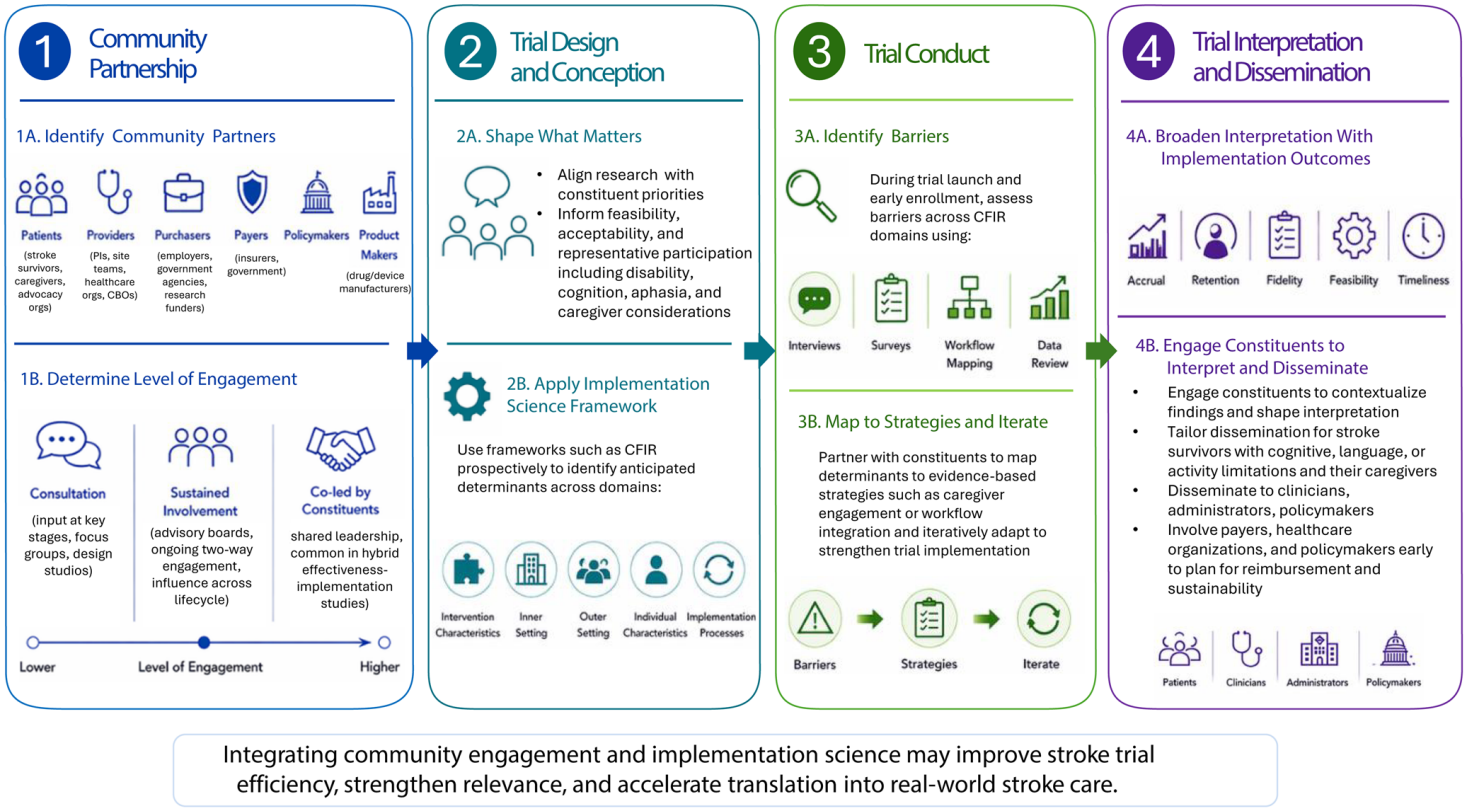
A framework for integrating community engagement and implementation science in stroke trials.

## 1 A Pragmatic Framework for Integrating Community Engagement and Implementation Science

The impact of clinical trials depends not only on what is tested, but also on how effectively trials are implemented within real‐world health systems and communities. Central to the proposed framework is the recognition that clinical trials themselves are implementation challenges requiring adoption by sites, integration into clinical workflows, participation by stroke patients and caregivers, and coordination across health systems [14, 15]. Community engagement helps ensure that trials address clinically meaningful questions and align with the needs and priorities of affected constituents, while implementation science offers structured approaches to identify multilevel determinants and select strategies to support trial feasibility, conduct, and translation.

## 2 Phase 1: Community Partnership

Operationalizing this approach begins with identifying which community partners are needed and determining their optimal level of engagement. Community partners for clinical trials may include patients (e.g., stroke survivors, stroke caregivers, and representatives from patient advocacy organizations), providers (e.g., site PIs, site teams, healthcare organizations, community‐based organizations), healthcare purchasers (i.e., employers, government agencies, research funders), payers (i.e., insurers, government), policymakers, and product makers (i.e., drug and device manufacturers) [16]. The composition of constituents varies based on stroke trials objectives, setting and broader context [17]. Acute, prevention, and recovery trials may rely on different constituencies. For example, an acute stroke trial may partner with Emergency Medical Services and the Emergency Department for recruitment, a clinical trial network to conduct the trial, and hospital administrators or payers to address feasibility, reimbursement, and downstream adoption [18]. In contrast, a recovery trial for chronic stroke survivors may partner with community‐based organizations for recruitment, and occupational therapists to develop the intervention, while also engaging with local government or insurers to ensure the intervention can be supported and sustained within the community‐setting [19].

In terms of level of engagement, we envision that most stroke clinical trials would benefit from community engagement through either consultation or sustained involvement (Table S1) [20]. Within community consultation, different constituents provide input at various stages of the trial lifecycle. Activities may include focus groups to understand the needs of stroke survivors or community members, trial design studios, and, for trials conducted under an exception from informed consent, community consultation and public disclosure [21]. Consultation is particularly suitable for acute stroke trials given narrow eligibility windows, protocol‐driven care, and the potential use of exception from informed consent [22]. In contrast, more sustained community involvement may be more suitable for prevention and recovery trials where a community advisory board could influence the trial through its lifecycle. Studies where the trial is co‐led by constituents are more frequently used in hybrid effectiveness–implementation studies such as those aimed at increasing acute stroke treatments [9, 23].

## 3 Phase 2: Trial Conception and Design

Integrating community engagement and implementation science early in trial conception may help strengthen study design and impact. Community partners can help shape the research question to ensure it reflects priorities that matter most to patients, caregivers, and communities. They can also inform feasibility, acceptability, and equity considerations that influence trial design from the outset [24].

Building on this early input, and as the trial moves into the trial design phase, implementation science frameworks such as the Consolidated Framework for Implementation Research (CFIR) can be applied prospectively by the research team to identify anticipated determinants that may influence trial conduct across intervention characteristics, inner and outer settings, individual characteristics, and implementation processes to mitigate barriers and strengthen facilitators (Figure S1) [25, 26, 27]. Prior efforts to address barriers in clinical trials have mostly focused on participant‐related barriers [28, 29]. The CFIR broadens this perspective by directing attention to a wider set of determinants which encourages a systematic evaluation of site‐, organizational‐, and system‐level barriers in addition to participant‐level issues.

This early engagement and assessment of anticipated determinants can surface contextual barriers such as transportation constraints, caregiver burden, workflow pressures in emergency departments, reimbursement concerns, and health‐related social risks that may influence stroke survivors' participation or site adoption [7, 30, 31, 32]. These concerns highlight the inevitable trade‐offs in clinical trials. Many trial decisions lack a single “right” answer, yet these decisions directly affect participant experience, trial feasibility, cost, and future adoption. For example, trialists may prefer longer and more frequent in‐person outcome assessments to capture exploratory endpoints, whereas participants may prioritize shorter, less frequent remote assessments due to transportation barriers and time constraints. Similarly, trial teams may design higher‐intensity interventions to maximize efficacy, whereas health systems and payers may prioritize simpler, lower‐cost approaches that can be more readily integrated and sustained in routine care. By surfacing these decisions and engaging constituents in the conception and design, stroke trials can more transparently navigate these trade‐offs and refine protocols in light of the real‐world constraints increasing the likelihood that they are feasible, equitable, and positioned for uptake into real‐world practice.

## 4 Phase 3: Trial Conduct

Building on this foundation, during trial conduct, community engagement and implementation science approaches support efficient rollout and ongoing trial modifications. Observations and assessments during trial launch and early enrollment via brief interviews, workflow mapping, surveys, or data review allow trial teams to identify emerging barriers across CFIR domains. In partnership with engaged constituents, these determinants can be mapped to evidence‐based implementation strategies (Table 1) [33]. For example, if enrollment is limited by emergency department workflow pressures, strategies such as identifying site champions, workflow redesign, or restructuring workflows to integrate study introduction into routine clinical care with handoff to the research team may be warranted. If retention is limited by caregiver burden or transportation challenges, adaptations such as remote assessments or flexible scheduling may be warranted. This iterative process strengthens trial efficiency while maintaining alignment with constituent priorities [34].

**TABLE 1:** Applying community engagement and implementation science across the stroke trial lifecycle.

Trial phase	CFIR domain	Common clinical trial barriers	Community partners	Implementation strategies (ERIC‐aligned)
Phase 1: Community engagement	Implementation process	Limited identification of partners; unclear roles; misaligned expectations	Patients/caregivers, clinicians, providers, purchasers, payers; policy makers, product makers	Identify and prepare champions; build a coalition; partner interrelationships; local needs assessment; advisory boards
Phase 2: Conception and design—	Intervention (trial protocol)	Protocol complexity; participant burden; consent challenges	Stroke survivors; caregivers	Adapt and tailor to context; simplify protocols and consent; promote adaptability
Phase 2: Conception and design—Outer setting	Outer setting (Trial network, funders, industry sponsors, structural determinants of health)	IRB delays; funding misalignment; regulatory pressures; non‐medical factors	Regulators; funders; payers; administrators; community orgs; government	Promote network weaving; access new funding; implementation blueprint; policy advocacy; align incentives
Phase 3: Trial conduct—Inner setting	Inner setting (Trial sites, resources, clinical structures)	Limited resources; staffing shortages; competing demands	Site teams; clinical leaders; community orgs	Provide ongoing consultation; implementation facilitation; change workflow integration; restructure workflows; ongoing training and education
Phase 3: Trial conduct—Characteristics of individuals	Characteristics of individuals (site PI, coordinators, participants)	Limited experience; provider beliefs; patient demands; caregiver burden	Patients; caregivers; coordinators; rehab teams	Educational outreach; educational materials; tailor strategies; external facilitation; incentives; reduce participation barriers
Phase 4: Interpretation and dissemination	Implementation process	Poor messaging; workflow mismatch	Patients; caregivers; clinicians; community orgs; systems; policymakers	Dissemination materials; mass media; champions; adapt to context; integrate into systems

## 5 Phase 4: Interpretation and Dissemination

Extending this approach beyond trial conduct, integration of community engagement and implementation science continues into interpretation, dissemination, and translation. Implementation science supports interpretation beyond efficacy endpoints to include trial implementation outcomes such as penetration and sustainability (recruitment and retention), fidelity (adherence to trial procedures as intended), feasibility (ability to successfully meet enrollment and trial conduct goals), and timeliness (reasonable progression of enrollment and trial completion) [15, 35]. Engaged constituents can inform interpretation of findings, contextualize results, and shape dissemination strategies tailored to multiple audiences, including stroke survivors with cognitive, language, or activity limitations, caregivers, clinicians, administrators, and policymakers [9]. Early involvement of payers, healthcare organizations, and policymakers may facilitate planning for reimbursement and sustainability if findings are practice‐changing. Once efficacy and safety have been established, hybrid effectiveness–implementation designs provide an opportunity to simultaneously evaluate clinical outcomes and implementation strategies, accelerating adoption into routine stroke care [36]. Such study designs have been used in stroke primary prevention [37], acute stroke treatment [38], and are emerging in posthospital stroke services [39].

## 6 Limitations and Conclusion

One limitation to our proposed pragmatic framework is that maintaining constituent engagement requires sustained resources, in the setting where resources and funding are often limited [40, 41]. Concerns have also been raised about tokenistic engagement where feedback is solicited from constituents but not incorporated into trial decisions due, in part, to lack of resources [41]. Finally, there is limited evidence comparing the effectiveness of the different levels of constituent engagement. Rigorous evaluation of these approaches would require randomizing trials to distinct engagement strategies.

In summary, we propose that integrating community engagement with implementation science offers a pragmatic approach to addressing the inherent challenges in conducting stroke clinical trials. By aligning trial design and conduct with the needs and priorities of the constituents affected by stroke and instituting implementation strategies to address multilevel barriers in clinical trials, this framework has the potential to strengthen trial efficiency, clinical relevance, accelerate translation into real‐world practice, and advance stroke health for all [42].

## Author Contributions

L..E.S. conceptualized the study (lead), drafted the manuscript, and created the visualizations. D.L.B. and B.B.‐A. contributed to study conceptualization (supporting role) and provided critical revisions to the manuscript.

## Funding

This project was supported, in part, by NIH/NINDS (U01NS086872), NIH/NINDS (R01NS128072), and National Center for Advancing Translational Sciences (UM1TR00512). The content is solely the responsibility of the authors and does not necessarily represent the official views of the National Institutes of Health.

## Conflicts of Interest

The authors declare no conflicts of interest.

## Supporting information


**Figure S1:** Determinants of clinical trial implementation based on the CFIR.
**Table S1:** Forms of constituent engagement in clinical trials.

## Data Availability

No datasets were generated or analyzed during the current study.
