# Supplementary material for: Advancing Stroke Clinical Trials Using Community Engagement and Implementation Science Approaches
Source: Ann Clin Transl Neurol. 2026 Jun 25:10.1002/acn3.70461. Online ahead of print. doi: 10.1002/acn3.70461 (PMC13394125; doi:10.1002/acn3.70461)
Supplement: Supplementary file 1 — Figure S1: Determinants of clinical trial implementation based on the CFIR. Table S1: Forms of constituent engagement in clinical trials. [file ACN3-9999-0-s001.docx]

Supplementary Figure 1: Determinants of clinical trial implementation based on the CFIR


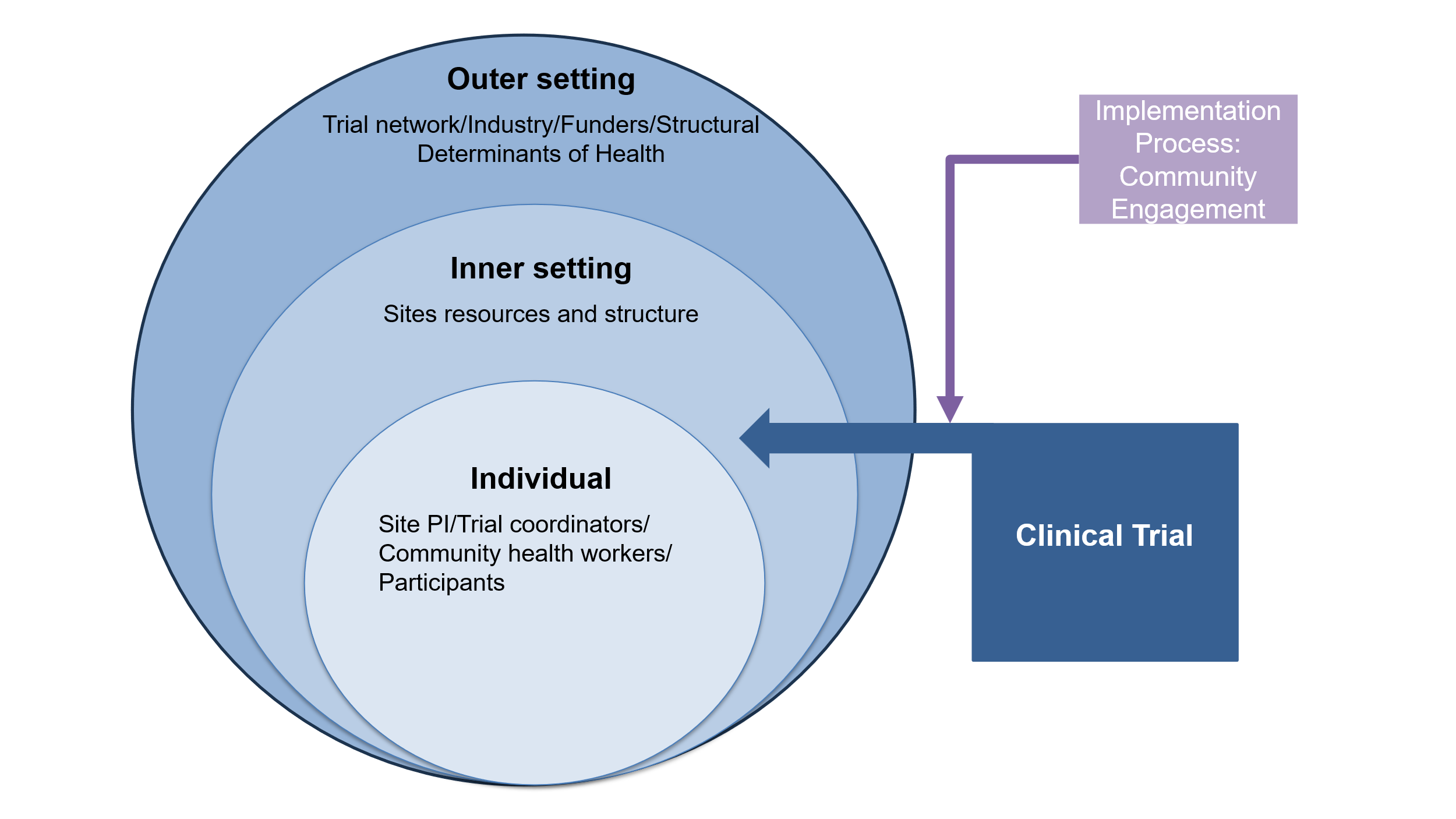


Supplementary Table 1: Forms of Constituent Engagement in Clinical Trials

|  | Inform | Consult | Involve | Collaborate | Community-led |
| --- | --- | --- | --- | --- | --- |
| Description | Outreach | One-time interaction | Ongoing trial influence | Shared-decision making | Community governed |
| Engagement | Constituents made aware of the trial | Constituents advise on the trial components | Constituents provide sustained input throughout the trial | Constituents and trialists co-lead the trial | Constituents lead the trial |
| Example Activities | Community presentations about the trial  Study websites | Focus group to understand community needs  Design studio to refine recruitment materials  Community consultation  Public disclosure | Standing community advisory board influences throughout the trial lifecycle  Embedded constituents on trial committees (e.g protocol, recruitment/retention, dissemination etc) | Co-design trials  Constituents as co-investigator and co-authors | Community dissemination and implementation studies  Trials embedded in community organizations with trialists support |
| Constituent engagement intensity | Low | Low-moderate | Moderate | High | Very High |
| Constituent decision-making power | None | Advisory | Influence without authority | Shared authority | Final decision authority |
